# Simplified models of aerosol collision and deposition for disease transmission

**DOI:** 10.1038/s41598-023-48053-0

**Published:** 2023-11-27

**Authors:** Sunghwan Sunny Jung

**Affiliations:** Department of Biological and Environmental Engineering, Cornell University, Ithaca, NY 14853 USA

**Keywords:** Fluid dynamics, Infectious diseases

## Abstract

Fluid-mechanics research has focused primarily on droplets/aerosols being expelled from infected individuals and transmission of well-mixed aerosols indoors. However, aerosol collisions with susceptible hosts earlier in the spread, as well as aerosol deposition in the nasal cavity, have been relatively overlooked. In this paper, two simple fluid models are presented to gain a better understanding of the collision and deposition between a human and aerosols. The first model is based on the impact of turbulent diffusion coefficients and air flow in a room on the collisions between aerosols and humans. Infection rates can be determined based on factors such as air circulation and geometry as an infection zone expands from an infected host. The second model clarifies how aerosols of different sizes adhere to different parts of the respiratory tract. Based on the inhalation rate and the nasal cavity shape, the critical particle size and the deposition location can be determined. Our study offers simple fluid models to understand the effects of geometric factors and air flows on the aerosol transmission and deposition.

## Introduction

There is a growing field of research on the fluid dynamics of disease transmission, which involves studying the physics of how fluids and aerosols flow in the context of infectious diseases^1^. There has been research focused on emphasizing aerosol ejection from an infected host^2–5^ or developing probability models to understand how diseases spread through contact between an infected source and a susceptible target, assuming that they are in a steady state^6,7^. However, the localized inception and deposition of pathogen-laden aerosols on a susceptible host have not received much attention compared to other aspects of disease transmission.

The well-mixed theory is a model widely used to predict the transmission of diseases in enclosed spaces^6,7^. It is based on the assumption that disease-causing agents are uniformly distributed throughout the space. However, the well-mixed model has limitations, particularly in predicting the concentration of the disease-causing agent near the infected person^8,9^. However, aerosol diffusion by turbulent flows is non-uniform and the collision/inception rate is highly localized. Hence, the well-mixed model can be improved with a better characterization of the concentration of pathogen-laden aerosols.

Turbulent diffusion can decrease the concentration of pathogen-laden aerosols over time, as the aerosols are transported away from the infected person^10^. Hence, well-mixed models may be unable to accurately capture short-term and localized concentrations of disease-causing aerosols. The turbulent diffusion coefficient is a measure of the rate of turbulent diffusion, which is an important factor in determining the concentration of pathogen-laden aerosols^11,12^. Unlike constant molecular diffusion, the eddy/turbulent diffusivity depends on air speed and characteristic length. Turbulent diffusion diffusivities are measured under various external conditions such as air flow speed and environmental conditions indoors to calculate a reliable estimate of the turbulent diffusion coefficient^13–17^.

A kinetic collision process involves the interaction between two or multiple particles that leads to a chemical reaction or adhesion by collision^18^. Especially, in an inelastic collision, the total kinetic energy is not conserved, and the particles may stick together or form a single particle after the collision. The inelastic collision between two particles can be analyzed by considering the densities and velocities of the particles and the ambient conditions. Collisions between particles and bubbles are important in mining flotation because they influence particle interaction, attachment, and detachment. The flotation process relies on this kinetic collision process to separate minerals from the ore^19,20^.

Geometry plays a crucial role in understanding aerosol deposition in the nasal cavity^21^. The nasal cavity is curved towards the nasopharynx to allow for the air passage from the nostril to the throat. The nasopharynx is located behind the nose and above the back of the throat. As a result of the curvature of the nasal cavity, large aerosols ($\gtrsim$ 5 µm) are more likely to be deposited in the beginning nasal cavity during inhalation. In particular, the highly tortuous air passage in animal noses can influence aerosol deposition^22,23^. Cheng et al.^24^ demonstrated that previous experimental measurements have established a simple relation between an impaction parameter and deposition probability. Numerical simulations have shown that the movement and deposition of aerosols in the nasal cavity are significantly affected by changing flow and aerosol size. The effect of nasal cavity morphology on the distribution patterns of inhaled aerosols remains less investigated through a simple fluid model except for Ref.^24^. Moreover, it can help us understand why certain aerosols will deposit in certain areas of the nasal cavity and why some aerosols may be more harmful than others.

This paper proposes two simple fluid-mechanics models of airborne infection to address several limitations mentioned above. The first model is based on the collisions between humans and aerosols that are caused by turbulent diffusion coefficients and air circulation in a room. In this way, infection rates can be explained in terms of air circulation and other geometric parameters. The second model describes how aerosols of different sizes attach to different parts of the respiratory tract based on their size. Based on the inhalation rate as well as the shape of the nasal cavity, the critical aerosol size and deposition location will be determined.

## Results

### Aerosol collision

To understand how disease is transmitted through aerosols, it is important to understand how aerosols are collided with humans when strong air currents are present indoors. The collision of two different entities in turbulence has also been the subject of several studies. For example, understanding bubble-particle collisions in turbulence has practical applications in various processes such as froth flotation, which is used in mineral processing and other phase-separation processes^19,25,26^. Generally, the density, size, and speed of aerosols and humans determine the likelihood of collision^3,27^. To translate the bubble-particle model to the human disease infection model, we consider the number density of healthy humans (*N*_1_) and aerosols (*N*_2_). Accordingly, the equation of healthy humans can be expressed as follows: assuming that *N*_2_ >> *N*_1_ and *N*_2_ remains almost constant, the process may be treated as a pseudo first-order process as

1 $$\frac{d{N}_{1}}{dt}=-{Z}_{12},$$

where $t$ is time, ${Z}_{12}$ is the number of collisions between humans and aerosols per unit volume per time, and *N*_*i*_ is the number density of the $i$th object. As in Refs.^18,28^, the collision probability is estimated by multiplying the collision diameter with the number densities, and the relative velocity between human and infectious aerosols.

2 $${Z}_{12}\approx {N}_{human}\,{N}_{aerosol}{d}_{12}^{2}{U}_{12}.$$

Here, *d*_12_ is the collision diameter (sum of effective radii of humans and aerosols), and *U*_12_ is the relative velocity between humans and infectious aerosols, respectively. Due to the aerosol’s smaller diameter than the nostril, the collision diameter can be approximated by the nose size. During inhalation, aerosols enter the respiratory tract and deposit on the mucosal surface, leading to infection. Since the human inhalation speed can be higher than the ambient air speed or the aerosol speed, *U*_12_ can be assumed to be the inhalation air speed, *U*_*inhale*_. Therefore, *d*_12_^2^* U*_12_ represents the inhaling flow flux, *Q*_*inhale*_.

**Figure 1:**
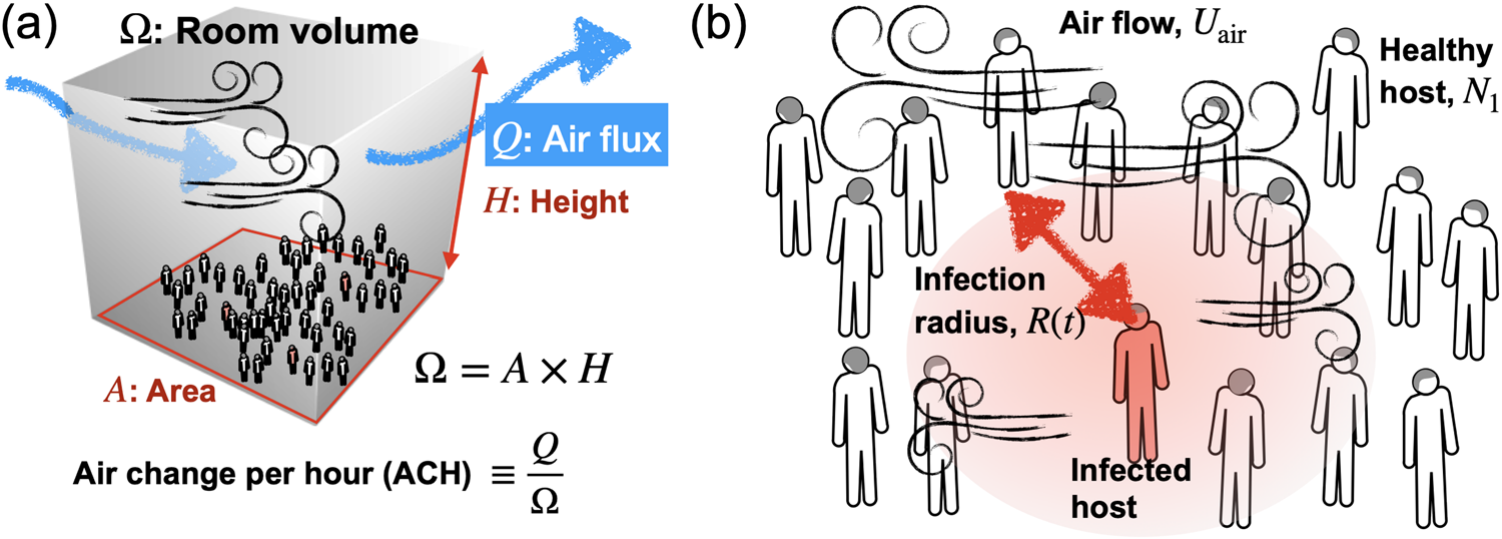
(**a**) Air flow in an enclosed space. There is a height of *H* and an area of *A* in the room, so the total volume of the room is Ω. A ventilation system generates a flow of air called *Q*. An air change per hour, ACH is calculated by dividing the air flux by the volume of the room. (**b**) There would be an infected person and many healthy people in the room. Infection aerosols are spread by air flows (its speed, *U*_*air*_) around the infection radius, *R(t)*. *N*_1_ refers to the number density of healthy people.

Air changes per hour (ACH) is an important factor in characterizing air flows and controlling airborne disease transmission. ACH also provides a dilution factor for possible infectious agents; increasing ventilation airflow rate can dilute concentrations when the contaminant source is constant^9,29,30^. As shown in Fig. 1a, ACH is defined as the number of times the air in a space is replaced in an hour as

3 $$ACH=\frac{Q}{\Omega },$$

where *Q* is the air flow rate and Ω is the volume of the room (height times area of the room). It is recommended to maintain a higher ACH to reduce the risk of airborne transmission of infectious diseases. ACH plays a significant role in infection transmission and disease control. The characteristic air speed for a given space is the flow rate *Q* divided by the area *A* of the space as ${U}_{air}=\frac{Q}{A}=\frac{Q}{A\cdot H}H= ACH \cdot H$.

The Reynolds number is a dimensionless quantity used to determine the type of flow pattern as laminar or turbulent. It is defined by the ratio of inertial forces to viscous forces. The Reynolds number is calculated as

4 $$Re=\frac{{U}_{air}H}{{\nu }_{air}}\simeq \frac{ACH\cdot {H}^{2}}{1.5 \times {10}^{-5} {\text{ m}}^{2}/{\text{s}}}.$$

The ceiling height is assumed to be about 3 m, so the corresponding Reynolds number can be estimated solely based on ACH. At an ACH of 5 h^−1^, the Reynolds number is generally about 800, which is typical for residential buildings. At an ACH of 30 h^−1^, the Reynolds number is approximately 5000, which is typical for bio-safety and highly specialized laboratories.

The infection radius of the source expands with turbulence, which increases the spread of aerosols as shown Fig. 1b. This means that the aerosols will spread over a larger area and the number density of the aerosols, N_2_ will be lower. The turbulent diffusion coefficient is used to predict the mean and dispersion of concentrations. The higher the ACH, the faster the aerosols spread and the lower the concentration in any given area. The turbulent diffusion coefficient, *D* is related to the ACH in an indoor environment as *D* = ACH* h*^2^^13^. We simplify that the diffusion radius is equal to its original size plus the square root of the turbulent diffusion coefficient (*D*) times the time (*t*) as *R(t)* = *R*_0_ + *(Dt)*^1/2^.

Temperature and relative humidity affect aerosol evaporation^31–33^. Higher relative humidity or lower temperature can inhibit evaporation and lead to a change in the size distribution of aerosols. For a simple model to estimate infection radius, we will neglect the effect of relative humidity and temperature. Then, the mass conservation shows

5 $$\frac{{N}_{2}(t)}{{N}_{2}^{0}}={\left(\frac{{R}_{0}}{{R}_{0}+{\left(Dt\right)}^{1/2}}\right)}^{3}= {\left(\frac{1}{1+{\left(ACH ({h/{R}_{0})}^{2}t\right)}^{1/2}}\right)}^{3}.$$

We assume that an individual is very likely to be infected by more than 1% of initial doses of pathogens. For simplicity, the number of initial pathogen-laden droplets is set to be 1000. Hence, a new host needs at least 10 aerosols to become infected. In addition, it is assumed that the characteristic length *h* and the aerosol length *R* are, respectively, 1 mm and 100 µm. The critical time to reach to 1% of initial doses is calculated from Eq. (6) as $T={\mathrm{ACH}}^{-1}{(100\, \mathrm{\mu m}/1\, \mathrm{mm})}^{2} {\left({100}^{1/3}-1\right)}^{2}$.

**Figure 2:**
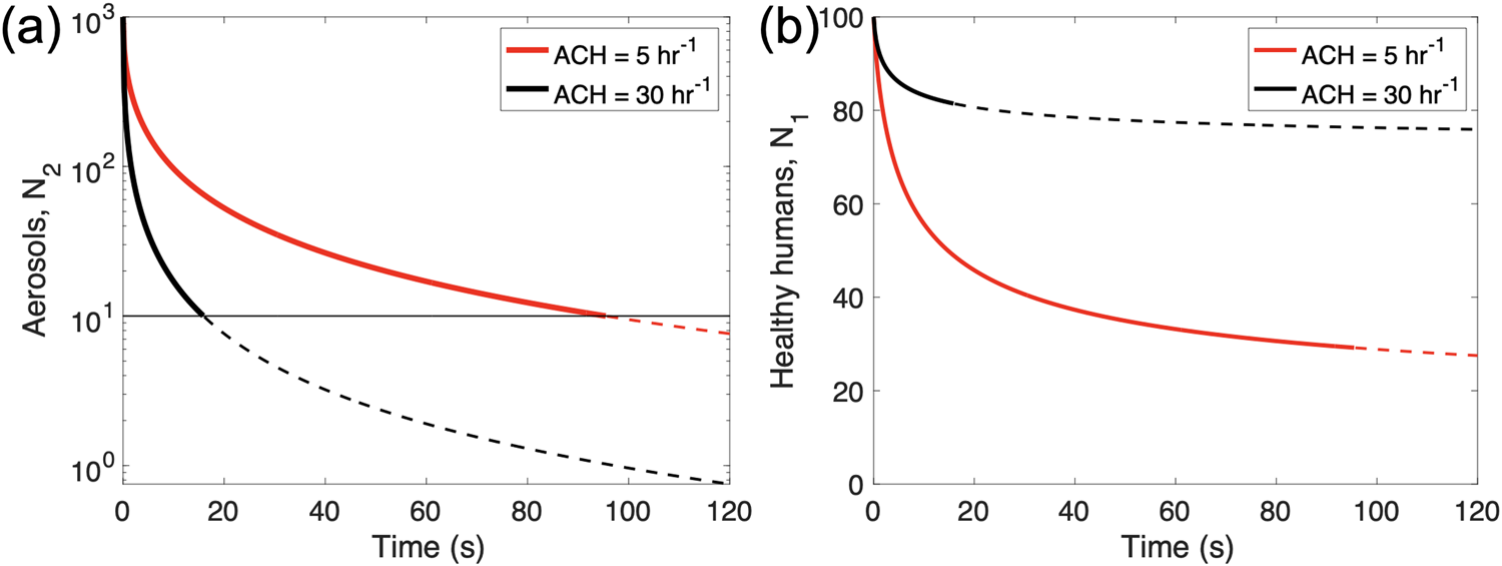
(**a**) The number density of aerosol, N_2_ vs the spreading time. We tested two ACH values; 5 and 30 h^−1^. It is assumed that numbers below 1% of the initial dose are ineffective, with an initial number density of 1000. Dotted lines indicate non-infectious aerosol doses. (**b**) The number density of healthy human, N_1_ vs the spreading time. With ACH = 30 h^−1^, only 20% of healthy people get infected. The aerosols from the infected person get dispersed quickly within 20 s to reach a non-infectious number density. With ACH = 5 h^−1^, about 70% of healthy people get infected.

A higher ACH will result in a shorter critical time as a result. For example, when ACH is 30 h^−1^, the critical time becomes only 16 s whereas ACH = 5 h^−1^ gives 95 secs as shown in Fig. 2a.

Finally, the kinetic equation for the number of healthy humans, Eq. (1) becomes

6 $$\frac{d{N}_{1}}{dt}=-{{N}_{1}N}_{2} {Q}_{inhale}.$$

By integrating it once from the initial time, one gets

7 $$\int \frac{d{N}_{1}}{{N}_{1}}=-{N}_{2}^{0}{Q}_{inhale}{\int }_{0}^{t}{\left(\frac{1}{1+{\left(ACH ({h/{R}_{0})}^{2}\tau \right)}^{1/2}}\right)}^{3} d\tau =-{N}_{2}^{0}{Q}_{inhale}\frac{t}{{\left(1+{\left(ACH ({h/{R}_{0})}^{2}t\right)}^{1/2}\right)}^{2}}.$$

As a result, the number of healthy humans in the room will decrease as

8 $${N}_{1}(t) ={N}_{1}^{0}\mathrm{exp}\left(-{N}_{2}^{0}{Q}_{inhale}\frac{t}{{\left(1+{\left(ACH ({h/{R}_{0})}^{2}t\right)}^{1/2}\right)}^{2}}\right) .$$

Figure 2b shows the number density of healthy humans according to different values of the ACH. At an ACH of 30 h^−1^, only 20% of healthy humans will get infected. At ACH = 5 h^−1^, 70% of the people in the room could become infected. In general, the rate of infection is lower if the ACH is higher, meaning that less people will become infected if the air in the room is properly circulated. This highlights the need for proper ventilation and air circulation to prevent the spread of disease.

### Aerosol deposition in respiratory tract

The fluid mechanics of aerosol deposition in the process of human disease transmission is so complex that it depends on various factors such as the size and velocity of droplets, environmental conditions, and the anatomical structure of respiratory tract. In this section, we will develop a model to predict aerosol deposition in the nasal cavity only.

Aerosol deposition on the wall is a topic in fluid mechanics that involves understanding how aerosols deposit on them. There are various models and techniques used to study this phenomenon, including analytical models, numerical simulations, and experimental studies.

By neglecting the Basset history term in the Maxey–Riley equation, particle motion in fluids can be written as

9 $${\rho }_{ptl}\frac{\pi {d}_{ptl}^{3}}{6}\frac{d\bf{u}_{ptl}}{dt}=3 \pi {\mu }_{air}{d}_{ptl}\left(\bf{U}_{inhale}-\bf{u}_{ptl}\right),$$

where ${\bf U}_{inhale}$ is the air velocity and ${\rho }_{ptl}$, ${d}_{ptl}$, and ${\bf u}_{ptl}$ are the density, aerodynamic diameter, and velocity of aerosols, respectively.

**Figure 3:**
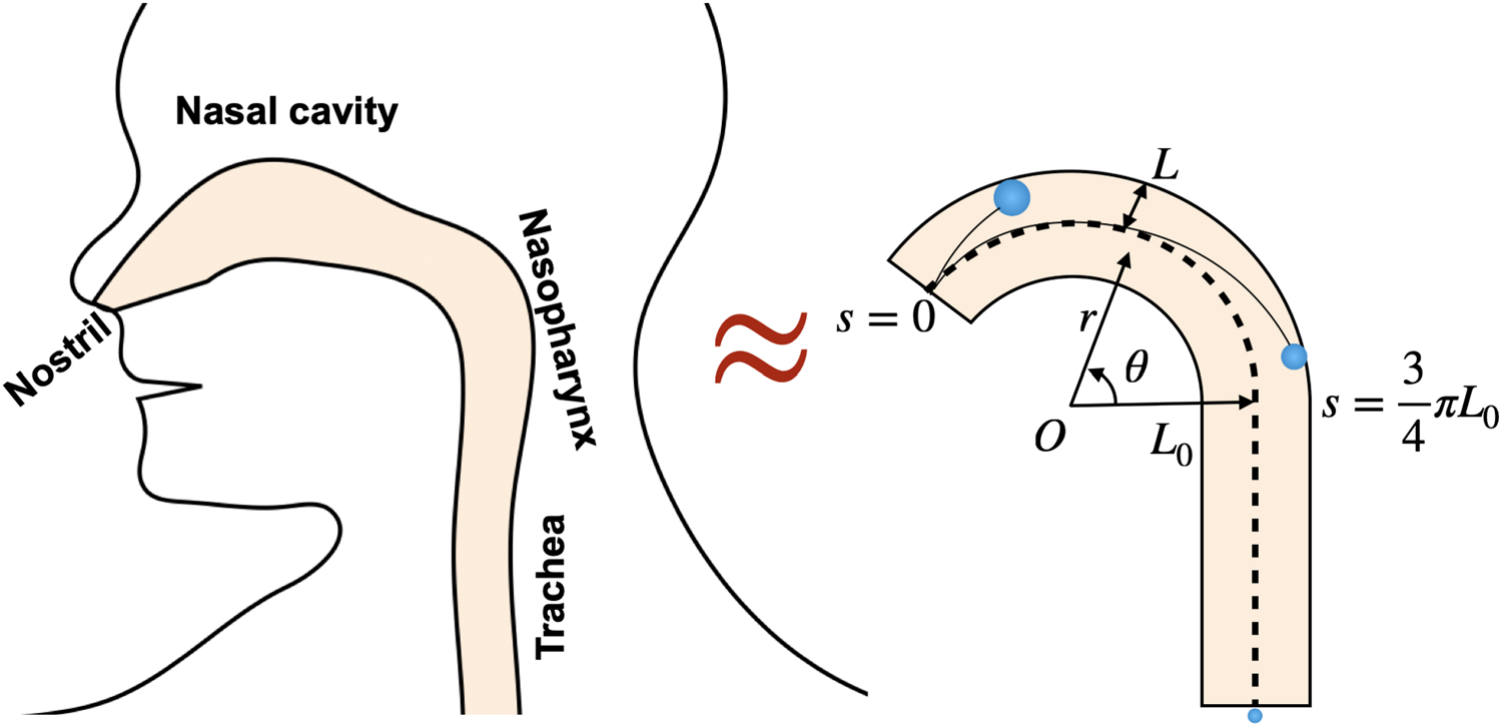
This schematic illustrates the structure of the nasal cavity and other respiratory tracts in the human body. The nasal cavity is curved from the nostril and the nasopharynx, but the later respiratory tract is quite straight downward. The respiratory tract can be approximated as a 270-degree curved pipe with a straight pipe at the end.

Stokes number is a dimensionless number that characterizes the behavior of particles suspended in a fluid flow. It is defined as the ratio of the characteristic time of a particle to a characteristic time of the flow or of an obstacle as $Stk\equiv \frac{{\rho }_{ptl}{d}_{ptl}^{2}}{18{\mu }_{air}}\frac{{U}_{inhale}}{{L}_{0}}$ where ${\mathbf{U}}_{inhale}$ is the air speed $({U}_{inhale}= | {\bf U}_{inhale} |)$ and *L*_*0*_ is the characteristic length. As shown in Fig. 3, we can consider *L*_*0*_ to be a radius of curvature of the nasal respiratory tract. The Stokes number allows us to estimate the behavior of aerosols in a fluid flow, which is affected by various factors such as aerosol size, fluid viscosity, and flow velocity.

Rewriting the governing equation for aerosols as

10 $$\frac{{\rho }_{ptl}{d}_{ptl}^{2}}{18{\mu }_{air}}\frac{d{\bf u}_{ptl}}{dt}\equiv \alpha \frac{d{\bf u}_{ptl}}{dt}=\left({\bf U}_{inhale}-{\bf u}_{ptl}\right),$$

where the characteristic time, $\alpha$ is given as ${\rho }_{ptl}{d}_{ptl}^{2}/18{\mu }_{air}=Stk\, ({L}_{0}/{U}_{inhale})$.

Here, the air velocity along a curved channel is given as ${\bf U}_{inhale}={U}_{inhale}\widehat{\theta }$ and the aerosol motion can be in both azimuthal and normal directions as ${\bf u}_{ptl}={v}_{\theta }\widehat{\theta }+{v}_{r}\widehat{r}$. The acceleration of the aerosol is given as $\frac{d{\bf u}_{ptl}}{dt}=\frac{d{v}_{\theta }}{dt}\widehat{\theta }+\left(\frac{d{v}_{r}}{dt}-{U}_{inhale}^{2}\kappa \right)\widehat{r}$ where $\kappa$ is given as a constant, $1/{L}_{0}$. A force balance in the radial direction is given as $\alpha \left(\frac{d{v}_{r}}{dt}-\frac{{U}_{inhale}^{2}}{{L}_{0}}\right)={-v}_{r}$ $\to \frac{d{v}_{r}}{dt}=-\frac{{v}_{r}}{\alpha }+\frac{{U}_{inhale}^{2}}{{L}_{0}}$.

With the boundary condition as ${v}_{r}\left(s=0\right)=0$, we get a solution as

11 $${v}_{r}\equiv \frac{dr}{dt}=\alpha \frac{{U}_{inhale}^{2}}{{L}_{0}}\left[1-\mathrm{exp}\left(-t/\alpha \right)\right].$$

Then, we can calculate the aerosol’s radial distance along the arc by integrating the radial velocity of the aerosol. The radial position of the aerosol over time is given as

12 $$r\left(t\right)={L}_{0}+\alpha \frac{{U}_{inhale}^{2}}{{L}_{0}}\left[t+ \alpha \left(\mathrm{exp}\left(-t/\alpha \right)-1\right)\right].$$

As soon as the radial travel distance equals or exceeds *L*_0_ + *L*, then the aerosol will deposit on the wall of the respiratory tract. As a result of this, the criterion of aerosol deposition becomes

13 $$\alpha \frac{{U}_{inhale}^{2}}{{L}_{0}}\left[t+ \alpha \left(\mathrm{exp}\left(-t/\alpha \right)-1\right)\right]>L.$$

**Figure 4:**
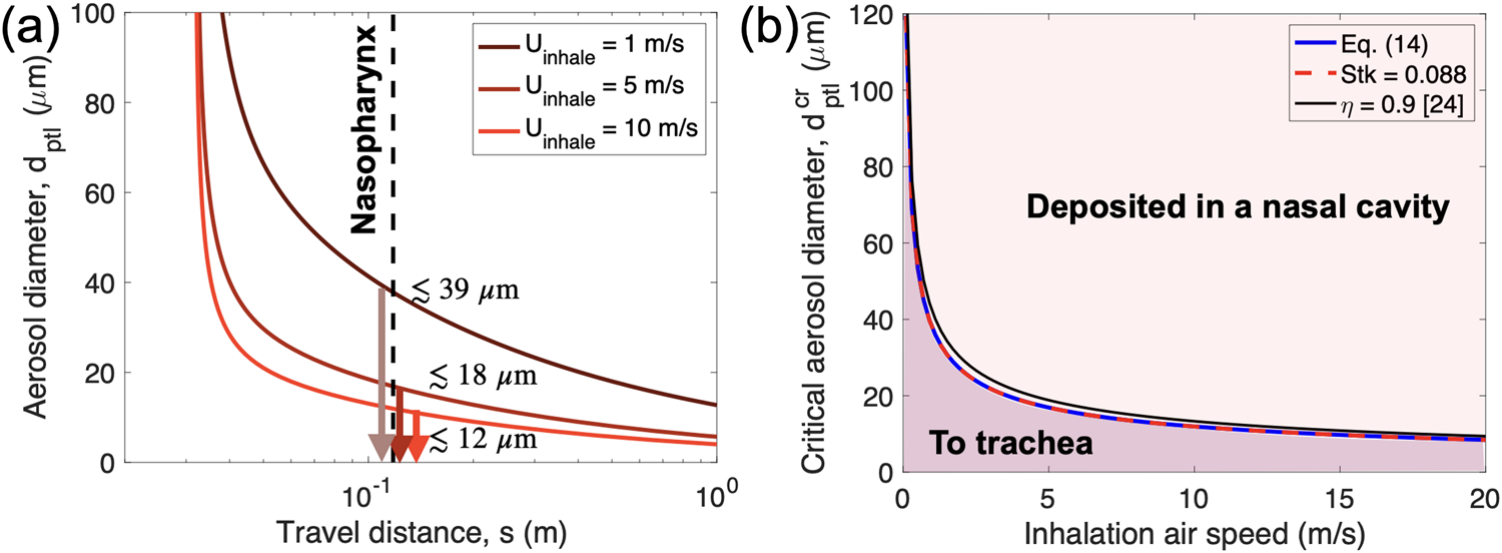
(**a**) The aerosol diameter vs its travel distance from the nostril. Here, the aerosol density is assumed to be 1000 kg/m^3^. The larger aerosols are deposited very close to the nostrils, whereas the smaller aerosols can travel further than the larger ones. Some aerosols pass the nasopharynx at approximate s = 0.11 cm. With *U*_*inhale*_ = 10 m/s (close to inhalation during exercise), aerosols only less than 12 µm will pass the nasopharynx and get to the lung. With *U*_*inhale*_ = 1 m/s like a normal breathe, aerosols less than 39 µm will pass the nasopharynx. (**b**) The critical aerosol diameter passing the nasopharynx vs the inhalation speed. In the case of a faster inhalation speed, there will be less and only smaller aerosols reaching the deeper trachea. The solid line is numerically calculated from Eq. (14) and the dotted line is based on the Stokes number equaling 0.088.

We can solve the above equation numerically. Figure 4a shows the travel distance along the respiratory tract as a function of air speed, i.e. the inhalation speed. The smaller aerosol travel farther from the nostrils.

Inhalation air speed ranges from 0.1 to 15 m/s and is affected by various activities such as speaking, coughing, sneezing and normal nasal breathing. Normal speaking creates an inhalation air speed around 4–6 m/s^34,35^ while coughing and sneezing induce about 10–15 m/s^35,36^. Normal nasal breathing causes an even lower rate at 0.1–2 m/s^37,38^. For *U*_*inhale*_ = 1 m/s (i.e. gentle breathing), aerosols less than 39 µm will pass the nasopharynx and get into a deeper respiratory tract. For *U*_*inhale*_ = 10 m/s (i.e. coughing or sneezing), only aerosols less than 12 µm will pass the nasopharynx, but most aerosols will deposit in the nasal cavity. This critical aerosol size of around 10 µm aligns with findings from numerical simulations using the Multiple-Path Particle Dosimetry Model^39–41^.

Next, we will find the critical aerosol size to deposit in the nasal cavity. Our nasal cavity can be approximated as a curved tube angled at 135 degrees (i.e. 3/4*π*) and the nasopharynx is located at *s* = 3/4* π L*_0_. Then, it is possible to approximate the time it will take to reach the nasopharynx as 3/4* π L*_*0*_ divided by the speed of the air; $T=\frac{3}{4}\pi {L}_{0}/{U}_{inhale}=\alpha \frac{3}{4}\pi St{k}^{-1}$. Figure 4b shows the solution to the above equation by replacing *t* with *T*.

14 $$\left[\frac{3}{4}\pi Stk+ \left(\mathrm{exp}\left(-\frac{3}{4}\pi St{k}^{-1}\right)-1\right){Stk}^{2}\right]>\frac{L}{{L}_{0}}.$$

The solution shows the inverse relation between the critical aerosol size and air speed. In general, even though the inhalation speed is close to 20 m/s, aerosols of 10 µm or smaller are able to reach the deeper trachea. We found that this numerical solution is very close to the solution of Stk ~ 0.088 (in red dotted line). Another finding is that this numerical solution is almost identical to the solution of Stk ~ 0.088 (in red dotted line). It is possible to derive this conclusion under the assumption that Stk <  < 1. As a result, the above equation becomes

15 $$St{k}^{2}-\frac{3}{4}\pi Stk+\frac{L}{{L}_{0}}<0.$$

The solution to the quadratic equation is Stk = 0.088. This means that if the Stokes number is less than 0.088, the aerosol will travel all the way to the nasopharynx. Additionally, our model predictions are compared to the proposed simple relation (efficiency $=1-\mathrm{exp}\left(-0.00028\, {d}^{2}\,{Q}_{inhale}\right)$) in Ref.^24^, which is based on other findings, and good agreement is observed in Fig. 4b when considering a 1 cm nostril diameter (depicted by the black line).

## Conclusion and discussion

Previously, fluid mechanics research has primarily focused on droplet/aerosol ejection from infected hosts, as well as the well-mixed theory in indoor environments. The dynamics of localized collisions between aerosols and healthy hosts, as well as the deposition of aerosols inside the nasal cavity, have been relatively understudied. Our paper presents two simple fluid models to better understand human collisions with aerosols and aerosol deposition in a nasal cavity after inception. From the first model, we found that if the ACH in the room is higher, than the rate of infection will be lower, which means that if the air in the room is circulated properly, there will be a lower chance of someone becoming infected. In the second model, the curved flow in the nasal cavity will allow large aerosols to deposit on the nasal wall. The critical aerosol size can be predicted as Stk ~ 0.1.

This research highlights the importance of aerosol collision and deposition to understand airborne disease transmission. This is not limited to human disease transmission, but can be extended to animal and plant disease transmissions too. Aerosol collision and deposition are ubiquitous because they are the main mechanisms for the movement of pathogenic aerosols (e.g. fungal spores, bacteria) in the air^42–44^. These aerosols can carry pathogens that can spread diseases and cause epidemics, not just among humans, but also among animals and plants.

## Data Availability

The data presented in this study are available upon reasonable request. Requests for access to the data should be directed to Dr. Sunghwan Jung.
